# Green Tea Extract Preserves Neuromuscular Activation and Muscle Damage Markers in Athletes Under Cumulative Fatigue

**DOI:** 10.3389/fphys.2018.01137

**Published:** 2018-08-17

**Authors:** Álvaro S. Machado, Willian da Silva, Mauren A. Souza, Felipe P. Carpes

**Affiliations:** ^1^Applied Neuromechanics Research Group, Laboratory of Neuromechanics, Federal University of Pampa, Uruguaiana, Brazil; ^2^Physiology Research Group, Laboratory of Neurochemistry, Federal University of Pampa, Uruguaiana, Brazil

**Keywords:** endurance, fatigue, exercise recovery, polyphenols, *Camellia sinensis*

## Abstract

A main implication of cumulative fatigue is the muscle damage that impairs neuromuscular function and training adaptations. These negative effects may limit performance when athletes exercise in consecutive days. In this regard, antioxidant supplementation has gain popularity among athletes. Green tea supplementation has been advocated as a strategy to improve exercise recovery due to the activity of its catechins with high antioxidant and anti-inflammatory potential. Here we performed a triple blinded placebo control experiment to determine the effect of green tea extract (GTE) from *Camellia sinensis* on muscle damage, oxidative stress, and neuromuscular activity in athletes submitted to consecutive sessions of exercise and fatigue. Sixteen trained amateur male athletes were randomly assigned to a GTE supplemented (500 mg/day) or placebo group during 15 days. Effects of supplementation were tested during repeated trials of submaximal cycling at 60% of peak power output performed after a protocol for cumulative fatigue of knee extensors. Muscle damage and oxidative stress showed lower magnitudes in response to fatigue after GTE supplementation. Placebo group showed impaired neuromuscular activity and higher muscle damage and oxidative stress compared to the GTE group during the cycling trials under fatigue. In summary, GTE supplementation showed positive effects on neuromuscular function in response to a condition of cumulative fatigue. It suggests GTE supplementation may have potential to serve as a strategy to improve performance and recovery in conditions of cumulative exercise.

## Introduction

Muscle fatigue is considered a limitant of athletic performance (Abbiss and Laursen, 2005). Its origin is multifactorial (Enoka and Duchateau, 2008), but it has been accepted that fatigue involves ATP depletion, muscle damage, and increased production of reactive oxygen species (ROS) resulting in a condition of oxidative stress (Hultman et al., 1986; Noakes, 1987; Steinbacher and Eckl, 2015). In general, fatigue negatively affects force production, and in the case of cycling, pedal forces, power output, and cadence are impaired (Diefenthaeler et al., 2012). Repeated bouts of strenuous exercise lead to a condition of cumulative fatigue in which the capacity of force production reduces, and for quadriceps muscles the recovery of force production after fatigue may need up to 3 days (Stewart et al., 2008).

In addition to the acute effects of fatigue on performance, consecutive sessions of exercise under a fatigue state may result in poor performance during training sessions and competitions (Stewart et al., 2008; Rodríguez-Marroyo et al., 2017). Conditions of cumulative fatigue may also increase the risk of injuries (Shing et al., 2016) and promote negative psychobiological adaptations (Rodríguez-Marroyo et al., 2017). However, there are many situations in which athletes have no choice other than sustain the performance under fatigue. This is the case of ultra-marathons, trail running, cycling distance challenges, and professional or amateur cycling tours (Lucia et al., 2001; Rodríguez-Marroyo et al., 2017). Therefore, strategies to minimize the fatigue effects on performance of repeated bouts of exercise are of interest for both coaches and athletes. A plausible strategy to achieve this purpose is to promote a faster exercise recovery.

In this regard, supplementation with natural products has attracted interest of athletes from different competitive levels. Considering that fatigue and its effects on performance during repeated sessions of exercise have important participation of oxidative stress and muscle damage (Kyparos et al., 2007), there is a crescent interest in supplementation with antioxidants like the green tea extract (GTE) from *Camellia sinensis*. GTE is rich in polyphenols including epigallocatechin gallate, epicatechin, epigallocatechin, and epicatechin gallate, which result in a powerful antioxidant activity (Jowko, 2015; Schimidt et al., 2017). Previous studies showed that GTE supplementation might reduce oxidative stress (Sugita et al., 2016) and promote improvement in the maximal oxygen uptake during cycling to exhaustion (Richards et al., 2010). Furthermore, GTE can reduce muscle soreness resultant of eccentric exercise (Herrlinger et al., 2015) and decrease markers of muscle damage after eccentric exercise (da Silva et al., 2018), intense aerobic exercise (Kuo et al., 2015), and strength exercises (Herrlinger et al., 2015). Similar effects were not found when a single-dose of GTE was intake before intense muscle-endurance tests (Jowko et al., 2012). The effects described for GTE supplementation on muscle damage and oxidative stress suggest that GTE could be a valid strategy to preserve performance during repeated bouts of exercise leading to a cumulative fatigue. To the best of our knowledge, our study is the first to address this question.

The potential effect of GTE supplementation on performance under a fatigue state has important practical applications. For instance, amateur competitions can involve consecutive days racing without a proper time for recovery (Shing et al., 2007; Magrini et al., 2017), and among professional athletes the recurrent performance along several consecutive days is a common condition. Therefore, the main goal of our study was to determine whether GTE supplementation minimizes muscle damage and oxidative stress contributing to the preservation of neuromuscular function in trained athletes exposed to consecutive sessions of exercise leading to cumulative fatigue.

## Materials and Methods

### Participants

We performed a randomized triple blinded placebo control experiment. Upon start of the study, 22 healthy trained men were recruited, but 16 completed all the phases of the study and had the data included in the analysis. They were randomly assigned to an intervention (green tea, *n* = 8) or placebo group (*n* = 8).

Participants were enrolled in systematic competitive cycling and/or running training including at least three sessions/week and had exercised more than 5 h/week in the past 12 months. The competitive level included participation in state and national competitions. During the study participants were requested to avoid ingestion of any medicine or stimulants, and to keep their regular routine of training and diet. Participants were followed regarding any injury, abrupt changes in their training volume and/or intensity. They should inform the need to start any medical treatment during the entire experimental phase. Six participants were excluded due to these criteria and therefore we had eight participants in each group. Intervention group was supplemented with green tea extract and the control received capsules with placebo. **Table 1** describes the study participants.

**Table 1:** Characteristics of study participants.

Characteristic	Green tea extract (*n* = 8)	Placebo (*n* = 8)
Age, years	37 (10)	37 (7)
Body mass, kg	77 (8)	76 (10)
Height, cm	170 (1)	170 (1)
Training experience, months	88 (64)	59 (48)
Training frequency, hours per week	7.2 (1.1)	7.2 (1.4)

*Data are presented as mean (standard deviation)*.

### Experimental Design

Experiments started with the participants completing an incremental maximal cycling test to determine the individual peak power output (PPO). In the following days they performed submaximal cycling trials combined or not with sessions of knee extension exercise to fatigue.

**FIGURE 1:**
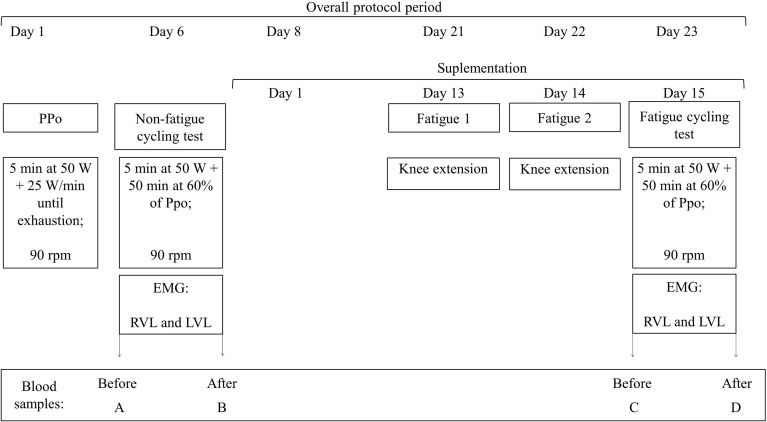
Experimental design. All participants completed the same protocols. Experiment started with the incremental maximal test to determine peak power output (PPO). Five days later, participants completed a submaximal cycling trial at constant load of 60% PPO, and the randomization of GTE and placebo supplementation was performed. In the last 3 days of supplementation participants repeated the submaximal cycling trial after two sessions of knee extensors exercise to fatigue. To avoid learning effects from the first test on the results after fatigue condition, half of the participants from each group performed the non-fatigue submaximal cycling test after the fatigue period, and the other half before. rpm: cadence, in revolution per minute, PPO: PPO, EMG: record of electrical muscle activation by surface electromyography, RVL: vastus lateralis from right leg, left vastus lateralis (LVL): vastus lateralis from left leg.

The whole experiment lasted 23 days for each participant. In the different visits to the laboratory, neuromuscular parameters were determined based on electrical neuromuscular activity; muscle damage and oxidative stress were determined from blood samples; and a cardiac monitor recorded heart rate. Data were compared between the GTE and placebo groups and between the conditions with or without the fatigue. All participants were evaluated with or without fatigue. In the case of non-fatigue condition, we ensured at least 5 days without supplementation and without performance of vigorous exercise (Chow et al., 2003, 2005), being at least 2 of the 5 days without performance of any exercise (Ahtiainen et al., 2003) before the laboratory tests. We call fatigue the condition of being tested after completing knee extension trials until exhaustion in two consecutive days before a submaximal cycling trial. **Figure 1** illustrates our experimental design. All participants signed a consent term before starting participation in the study; procedures were conducted in agreement with declaration of Helsinki, and this research was approved by the institutional committee of ethics in research (IRB no. 60376216.4.0000.5323).

### Cycling Protocols

Cycling trials were performed always between 3 and 6 pm on a cycle ergometer (Lode Excalibur Sport, Lode, Netherlands) properly adjusted to the individual body posture of the participants. The incremental maximal test for determination of PPO started with a 5 min warm up at 50 W and cadence of 90 rpm followed by increments of 25 W/min until the participant was no longer able to keep pedaling cadence higher than 70 rpm. The last workload completed was therefore named the PPO (Priego Quesada et al., 2016). The submaximal tests started with workload of 50 W during 5 min and then the workload was increased to 60% of the individual PPO (Priego Quesada et al., 2016), which was sustained for 50 min.

### Neuromuscular Assessment

Neuromuscular electrical activity was determined during the submaximal cycling tests using surface electromyography (EMG). EMG signals were recorded bilaterally from the vastus lateralis, which was selected due to its main role for power production in cycling (Bini et al., 2008). Data were sampled at 1.5 kHz using an EMG acquisition system (miniDTS and MyoMuscle, Noraxon, United States) following SENIAM guidelines for electrode placement and subject preparation (Hermens et al., 2000). EMG signals were filtered using a band-pass digital Butterworth filter with cut off frequency of 0.5–250 Hz. Onset and offset of neuromuscular electrical activity for each contraction burst were determined using the criteria of variation of two standard deviation for increase and decrease considering the average activation recorded during rest (Hodges and Bui, 1996). From each contraction burst during the cycling trials, the root mean square (RMS) value was determined as an indicator of magnitude of activation (Moritani et al., 1986) and the fast Fourier transform was computed to determine the median frequency, which was used as an indicator of fatigue (Cifrek et al., 2009). EMG signals were recorded alternating between 5 min of recording EMG (named “moment” 1–5, in the Section “Results”) and 5 min without recording. At the end, for each participant we had five moments of 5-min EMG record. EMG data from moment 1 (5–10 min of exercise) was considered the reference to the normalization of RMS values obtained during the exercise.

### Fatigue Condition

We aimed to elicit a condition of cumulative fatigue by combining 2 days of strenuous knee extension exercises until exhaustion and the performance of a submaximal cycling trial on the subsequent day. The trials for knee extension were also performed between 3 and 6 pm using a seated knee extensor machine with the participant performing concentric-eccentric knee extensions until exhaustion. The first set had the workload equivalent to 50% of the individual body mass. A metronome set at 20 beats per minute controlled the movement velocity. In the first set of repetitions the maximal number of voluntary repetitions was determined. After 30 s of rest, participants performed the second set aiming at a number of repetitions corresponding to 75% of the maximal number of voluntary repetitions performed in the first trial. It was repeated until participants were no longer able to perform more than 50% of the maximal number of voluntary repetition in the first trial. Cycling trial was performed 24 h after the second fatigue protocol.

### GTE Supplementation and Placebo

Participants received 15 capsules not identified and were advise to intake one capsule per day, before breakfast, with a glass of water. The capsules from GTE and placebo groups were identical. Supplementation was administrated in capsules because this strategy results in larger bioavailability (Henning et al., 2004). Capsules content were GTE and celulomax E, an inactive excipient that served as a placebo. GTE dose was defined considering the results from a previous study in which the same supplementation dose reduced fatigue-induced muscle damage (da Silva et al., 2018). GTE was purchased from a local commercial supplier, manipulated by a pharmaceutics registered professional, and tested using high performance liquid chromatography (HPLC) to ensure the presence of epigallocatechin gallate (1.60 mg/g), epicatechin (1.59 mg/g), epigallocatechin (16 mg/g), and epicatechin gallate (17.80 mg/g). HPLC was performed with a Shimadzu Prominence Auto Sampler (YL9100) system (Shimadzu, Kyoto, Japan), equipped with Shimadzu YL9110 reciprocating pumps connected to an YL9101 degasser with an YL9150 integrator, and YL9160 diode array detector. To determine compounds profile the extracts were analyzed using a reversed phase carried out under gradient conditions using Synergi Fusion-RP 80A column (4.6 × 250 mm). The mobile phase was composed of water (pH = 3): acetonitrile (5:95, v/v) in a gradient mode, until 35 min, in which the mobile phase was 100% acetonitrile. At 38 min water (pH = 3): acetonitrile (5:95, v/v) was used again, in isocratic mode, as a mobile phase, until 50 min. A flow rate of 0.8 mL/min was used and 20 μL of sample were injected. Phenolic compounds were identified and quantified by comparing the retention time and UV–Visible spectral data to known previously injected standards. The chromatography peaks were confirmed by comparing the retention time with those of reference standards and by DAD spectra. Calibration curves were determined for EGC (*y* = 101,79x - 10,283); EC (*y* = 91,872x + 7657); EGCG (*y* = 103,5x - 93,211); ECG (*y* = 112,17x - 81,22). All chromatography operations were performed at ambient temperature and in triplicate. During the supplementation period participants were requested to report any consume of stimulants, other supplements, medications, and teas originated from *C. sinensis* or other plant. Furthermore, they were requested to avoid consume of fruits, milk, caffeine, and alcohol on the day before each cycling tests when blood samples were collected (Sugita et al., 2016). Participants received daily messages to recall them about the orientations and to avoid mistakes in capsules intake.

### Blood Samples and Biochemistry Essays

Blood samples (10 mL) were collected from the ulnar vein before and after each cycling submaximal test. Samples were centrifuged (10 min, @3500 rpm) to separate the plasma that was stored at -80°C to further determination of total activity of creatine kinase (CK) (Noakes, 1987) using enzymatic commercial kits (Labtest). The blood samples for biochemical analyzes of oxidative damage were collected in tubes with heparin. The analysis of substances reactive to the thiobarbituric acid (TBARS) served to determine the lipid peroxidation (Ohkawa et al., 1979). To ensure that participants had no damage in soft tissues that could increase CK (for instance, a muscle strain, tendon, or ligament injury, etc.) we determined the serum levels of the C reactive protein (Pritchett, 1996) using immunological kits (Labtest). The blood analyses are named in the result section as: (A) pre cycling without fatigue, (B) post-cycling without fatigue, (C) pre cycling with fatigue, and (D) post-cycling with fatigue.

### Statistical Analyses

Data are expressed as mean and standard deviation. Normality of data distribution was confirmed using the Shapiro–Wilk test. EMG signals within cycling trials were compared between the moments by one-way ANOVA with Bonferroni *post hoc*, and the comparison between the groups and fatigue conditions by two-way ANOVA with Bonferroni *post hoc*. Biochemical and heart rate data were compared within cycling trials by one-way ANOVA with Bonferroni *post hoc*. For non-parametric data Friedman and Wilcoxon testes were used. Comparisons between the groups were performed using independent *t*-test. Significance level was set at 0.05 for all analysis.

## Results

Age (*t*_(1)_ = 0.457; *P* = 0.37), height (*t*_(1)_ = 0.514; *P* = 0.49), body mass (*t*_(1)_ = 1.016; *P* = 0.47), training experience (*t*_(1)_ = 1.576; *P* = 0.159), and training frequency (*t*_(1)_ = 0.242; *P* = 0.815) did not differ between GTE and placebo groups.

The PPO did not differ (*t*_(1)_ = 0.333; *P* = 0.123) between the GTE 303 (52) W and placebo 309 (49) W, which resulted in a submaximal cycling workload that did not differ (*t*_(1)_ = 0.333; *P* = 0.123) between the GTE 180 (27) W and placebo 181 (31) W.

### Biochemical Essays

Results of C-reactive protein suggest that participants from GTE and placebo groups did not suffer macro injuries related to the experiments (data not shown).

**FIGURE 2:**
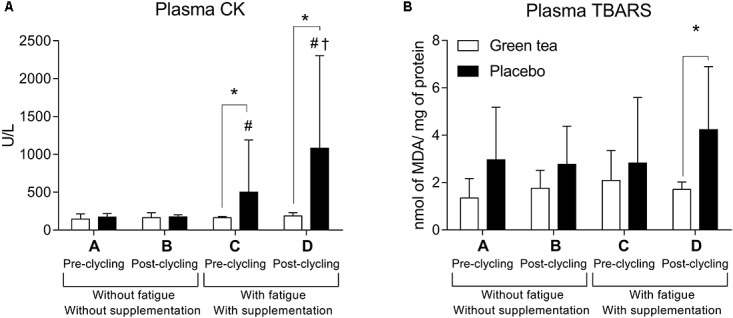
Plasma **(A)** creatine kinase (CK) and **(B)** plasma lipid peroxidation measured by thiobarbituric acid reactive substances (TBARS). ^∗^*P* < 0.05 between groups comparisons; ^#^*P* < 0.05 higher than A and B for the same group; ^†^*P* < 0.05 higher than C for the same group. MDA = Malondialdehyde.

Muscle damage was lower in the GTE supplemented participants. CK activity did not differ between GTE and placebo in pre cycling without fatigue (moment A, *F*_(1)_ = 1.600; *P* = 0.300) and post-cycling without fatigue (moment B, *F*_(1)_ = 1.890; *P* = 0.191). However, CK was higher in placebo pre cycling with fatigue (moment C, *F*_(1)_ = 19.496; *P* = 0.300), and post-cycling with fatigue (moment D, *F*_(1)_ = 18,917; *P* = 0,001). GTE supplementation protected against muscle damage as estimated by CK activity (*F*_(3)_ = 0.767; *P* = 0.522, **Figure 2A**), which did not happen in placebo group (*Z*_(1)_ = -2.100; *P* = 0.036).

Placebo group showed higher oxidative stress in the fatigue condition, suggesting a protective role of GTE supplementation. Oxidative stress assessed by lipid peroxidation (TBARS, **Figure 2B**) did not differ between the groups in moments A (*F*_(1)_ = 4.200; *P* = 0.600), B (*F*_(1)_ = 3.703; *P* = 0.075), and C (*F*_(1)_ = 0.522; *P* = 0.482). In moment D, TBARS was higher in the placebo than GTE group (F_(1)_ = 4.838; *P* = 0.045).

### Heart Rate

**FIGURE 3:**
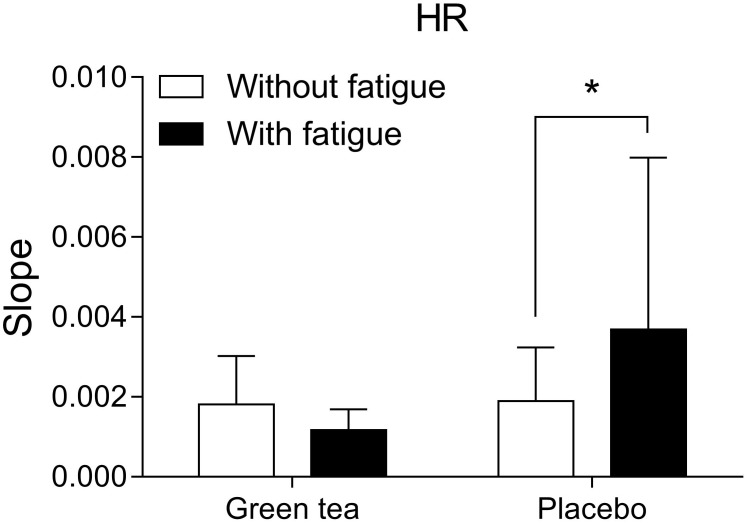
Heart rate (HR) curve slope over time of exercise in the different groups and conditions. ^∗^*P* < 0.05 between conditions.

Cardiovascular responses estimated by heart rate showed that GTE supplemented group experienced lower cardiac workload than placebo group. Heart rate responses to the cycling trials (**Figure 3**) were analyzed by the angular coefficient of the regression curve considering second-to-second data recorded during the exercise. Higher heart rate increase during exercise in the fatigue condition was observed in the placebo (*F*_(1)_ = 5.869; *P* = 0.030) compared to GTE group (*F*_(1)_ = 0.075; *P* = 0.788).

### Neuromuscular Activation

**FIGURE 4:**
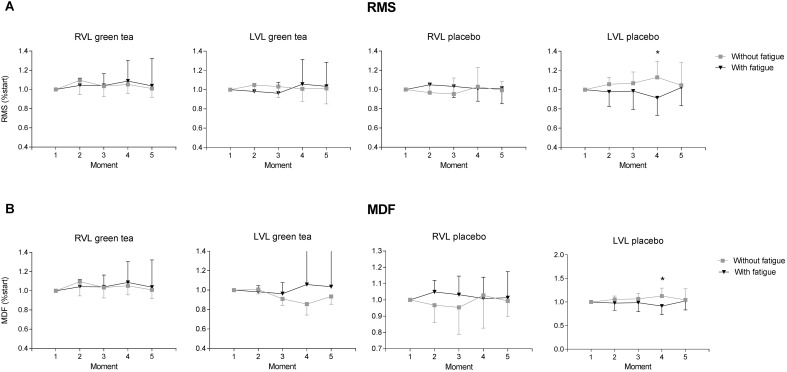
Results of neuromuscular electrical activity obtained from GTE and placebo groups during the cycling trials with and without fatigue condition. Data are shown as mean (bars) and standard deviation (vertical lines) for **(A)** root mean square (RMS) and **(B)** median frequency (MDF) normalized to the moment 1 for right (RVL) and left vastus lateralis (LVL). ^∗^*P* < 0.05 difference between conditions for the same group at a moment.

Neuromuscular activation from the left vastus lateralis (LVL) of the participants of placebo group showed significant impairment in the fatigue condition. Fatigue condition showed an effect for magnitude of neuromuscular activity estimated by the RMS values (**Figure 4A**) in the LVL that showed neuromuscular activation decreased by the end of the exercise in the fatigue condition in placebo (*F*_(4)_ = 5.510; *P* = 0.020) but not in GTE group (*F*_(4)_ = 2.151; *P* = 0.140). In the right vastus lateralis (RVL) fatigue condition did not showed an effect for magnitude of neuromuscular activity (**Figure 4A**) in both GTE (*F*_(4)_ = 0.009; *P* = 0.920) and placebo groups (*F*_(4)_ = 3.570; *P* = 0.060).

In GTE group, neuromuscular fatigue estimated by the median frequency during the submaximal cycling trial (**Figure 4B**) was not detected in the RVL (*F*_(4)_ = 0.009; *P* = 0.920) and LVL (*F*_(4)_ = 1.914; *P* = 0.170). Placebo group did not change median frequency of RVL (*F*_(4)_ = 0.680; *P* = 0.170) but showed a decrease in the LVL (*F*_(4)_ = 5.510; *P* = 0.020).

## Discussion

Here we set out to determine whether GTE supplementation could benefit performance under a condition of cumulative fatigue. GTE has been shown as a potential antioxidant, with positive effects on different tissues, and could be a good option for competitive sports. Despite of its popularity among athletes, few evidences of the benefits are available concerning amateur competitive sport. To the best of our knowledge, this is the first study demonstrating that GTE supplementation before cumulative fatigue minimizes muscle damage and oxidative stress in trained athletes, therefore playing a significant role in exercise recovery, and with important effects on neuromuscular and cardiovascular performance during exercise.

Previous studies on GTE supplementation in athletes were limited to the determination of performance improvement resultant of higher lipid oxidation due to GTE activity (Ichinose et al., 2011). Rather than an effect on energy supply, here we focused on performance during endurance trials of cycling under cumulative fatigue, which is close to the experienced by athletes in competitions lasting more than 1 day, and found GTE results supporting benefits of this supplementation on both muscle damage and recovery markers, as well neuromuscular function (Fuglevand et al., 1993). These are important implication for training and competition.

Placebo group showed higher muscle damage after fatigue. Increase in CK activity is commonly associated with damage resultant of mechanical stress and structural acute changes in the muscle, which happens in coexistence with increase in oxidative stress (Morillas-Ruiz et al., 2006). Such result supports the role of GTE in minimizing muscle damage resultant of exercise.

Oxidative stress is the most accepted explanation to the presence of muscle damage, and the results from GTE group support the lower oxidative stress as an explanation to the lower CK activity observed in the GTE supplemented group (Panza et al., 2008). CK activity determined from the circulating blood can be variable, and it is important to ensure the absence of other lesions that could influence CK activity. We found no changes in C-reactive protein and therefore attribute the changes in CK activity to the stress imposed by the exercise protocols (Pritchett, 1996).

Green tea extract supplementation resulted in stable lipid peroxidation, which was used as a marker of oxidative stress. It is known that oxidative stress is not cumulative along different days of exercise (Shing et al., 2007), but when we look at the oxidative stress data from the last session of cycling exercise we found lower oxidative stress in the GTE group. This result is in agreement with a previous study addressing sprints tasks that resulted in an oxidative stress condition in placebo but not in the GTE supplemented group (Jowko et al., 2015).

The exercise configuration used here leaded to an imbalance in the oxidative status resulting in oxidative stress (Vollaard et al., 2005). Oxidative stress has important implications on the contraction mechanisms and force output capacity (Prochniewicz et al., 2008). GTE catechins work as scavengers of reactive species of oxygen better than observed in response to other supplementation strategies commonly used in sports, such as vitamin C and E (Zaveri, 2006). It supports our idea that the antioxidant properties of GTE are the main explanation to our results in the fatigue condition.

The metabolic damage in the muscle tissue impaired muscle activation in a way visible considering simple markers of surface electromyography. Placebo group showed lower magnitudes of neuromuscular activity and higher indicators of muscle fatigue. The decrease in the magnitude of neuromuscular activation and its correlation with increase in markers of muscle fatigue is expected in muscles exposed to repeated bouts of exercise under cumulative fatigue (Mendez-Villanueva et al., 2012). Our cycling trials involved constant workload relative to the individual PPO. It doesn’t allow us to discuss decrement in force output as expected during a test for exhaustion (Diefenthaeler et al., 2012) because we fixed the mechanical load, but these results denote that our athletes were able to further recruit additional muscle fibers (Ericson et al., 1986) in order to sustain the exercise, and it had implications on the cardiovascular responses (Thomson et al., 2016), as we estimated by analyzing the heart rate data recorded during the cycling trials. While this rationale seems evident to the placebo group, GTE group showed better indicators of neuromuscular performance and cardiovascular demand.

Our study has limitations. We were unable to fully control the diet of the participants and it may have influenced the higher variability observed in the results, which is also a common issue in previous studies. To minimize this effect we delivered detailed recommendations to the participants, like avoiding intermittent fasting that affect oxidative stress (Dannecker et al., 2013). Also, we used the term “fatigue” despite of the difficulties to quantify this phenomena and the number of co-variables that can influence fatigue manifestations. We controlled the highest number of factors possible we could to minimize other variables of influence on our results. Neuromuscular results showed consistent impairments in the left leg, and it may have some relation with variables of motor control like leg preference, which deserves attention in future researches. Measurements of force would help to determine the extend of damage due to the exercise (McHugh et al., 1999). We were unable to evaluate knee extensors force. The pharmacokinetics of the catechins in the blood may have influenced our results. In rats dosed with green tea catechins, concentrations in the blood exhibited peak up to 3 h after intake (Janle et al., 2008). In humans, peak plasma concentrations are reached between 1.5 and 5 h depending on the catechin considered, but the variability and the level of metabolites are not clearly identified (Janle et al., 2008). We tried to minimize these effects by controlling the period of the day in which tests were performed according to the time when the supplementation was intake. Finally, although the dosage is different among the studies, a higher dosage is not related to better results on muscle soreness, for example (Arent et al., 2010; Rynders et al., 2014; Romain et al., 2017), and we selected a dosage that was shown to be effective in conditions of fatigue (da Silva et al., 2018).

## Conclusion

Green tea extract supplementation before an event of cumulative fatigue minimizes muscle damage and oxidative stress in trained athletes. It also shows positive effects on neuromuscular parameters related to muscle activation and muscle fatigue. Therefore, GTE supplementation can be considered a valid strategy in the context of competitive endurance sport aiming at exercise recovery and performance of athletes.

## Ethics Statement

This study was approved by the ethics committee from Universidade Federal do Pampa and all participants signed a consent term prior to start the participation in this research.

## Author Contributions

ÁM, WdS, MS, and FC designed the study, interpretated the data, and prepared the manuscript. ÁM, WdS, and MS collected and processed the data. All authors approved the final manuscript.

## Conflict of Interest Statement

The authors declare that the research was conducted in the absence of any commercial or financial relationships that could be construed as a potential conflict of interest.
